# The influence of DNA sequence on epigenome-induced pathologies

**DOI:** 10.1186/1756-8935-5-11

**Published:** 2012-07-20

**Authors:** Richard B Meagher, Kristofer J Müssar

**Affiliations:** 1 Genetics Department, Davison Life Sciences Building, University of Georgia, Athens, GA, 30605, USA

## Abstract

Clear cause-and-effect relationships are commonly established between genotype and the inherited risk of acquiring human and plant diseases and aberrant phenotypes. By contrast, few such cause-and-effect relationships are established linking a chromatin structure (that is, the epitype) with the transgenerational risk of acquiring a disease or abnormal phenotype. It is not entirely clear how epitypes are inherited from parent to offspring as populations evolve, even though epigenetics is proposed to be fundamental to evolution and the likelihood of acquiring many diseases. This article explores the hypothesis that, for transgenerationally inherited chromatin structures, “*genotype predisposes epitype*”, and that epitype functions as a modifier of gene expression within the classical central dogma of molecular biology. Evidence for the causal contribution of genotype to inherited epitypes and epigenetic risk comes primarily from two different kinds of studies discussed herein. The first and direct method of research proceeds by the examination of the transgenerational inheritance of epitype and the penetrance of phenotype among genetically related individuals. The second approach identifies epitypes that are duplicated (as DNA sequences are duplicated) and evolutionarily conserved among repeated patterns in the DNA sequence. The body of this article summarizes particularly robust examples of these studies from humans, mice, Arabidopsis, and other organisms. The bulk of the data from both areas of research support the hypothesis that genotypes predispose the likelihood of displaying various epitypes, but for only a few classes of epitype. This analysis suggests that renewed efforts are needed in identifying polymorphic DNA sequences that determine variable nucleosome positioning and DNA methylation as the primary cause of inherited epigenome-induced pathologies. By contrast, there is very little evidence that DNA sequence directly determines the inherited positioning of numerous and diverse post-translational modifications of histone side chains within nucleosomes. We discuss the medical and scientific implications of these observations on future research and on the development of solutions to epigenetically induced disorders.

## Review

### Cause-and-effect and epigenetic risk

The inheritance of numerous genetic risk factors for human and plant diseases as well as biotic and abiotic stress susceptibility phenotypes are well established [1-6]. Particular DNA mutations and their mechanistic effect on the timing, level, or quality of gene expression produce the risk of disease. Thus, a clear cause-and-effect relationship is established between the inherited aberrant genotype and the risk phenotype (that is, the increased chance or certainty of presenting a disease).

Epigenetics is cited as contributing to the risk of acquiring numerous diseases and aberrant phenotypes in human and plant populations based primarily on correlations between changes in chromatin structure and penetrance of the undesired phenotype [7-10]. There has been a growing suspicion, particularly since the 1980s, that - along with classical genetics - epigenetics is required to explain many complex phenotypes associated with disease [11,12]. The influences of age and environment (for example, chemicals, heat, nutrition, daylight) on various pathologies and the seemingly stochastic penetrance of developmental abnormalities are particularly difficult to interpret using purely molecular genetic models and are more easily explained by considering epigenetic control mechanisms [13-18]. However, few cause-and-effect relationships have been established that prove that that particular inherited cis-linked chromatin structures (epitypes) are in fact useful in predicting the inherited risk of acquiring disease phenotypes. Exceptions are the epigenetic silencing of the *skeletal-muscle ryanodine-receptor gene (RYR1)* that causes congenital myopathies and the *MutL Homolog 1 gene (MLH1)* that causes increased risk of colorectal or endometrial tumors, which are discussed in the following section.

Inherited risk epitypes should evolve in populations in ways similar to the evolution of genotypes [19]. The problem is that the transgenerational inheritance of epigenetic controls is not well understood in any multicellular organism and often difficult to prove. This is particularly true in humans and agricultural crops, where the need for understanding epigenetic risk is the greatest [20-27]. Without knowledge about the molecular basis for the transgenerational inheritance or generational reprogramming of defined epigenetic risk factors that contribute to disease, it is difficult to design effective targeted therapeutics for humans or to knowledgably alter breeding programs for crops that will avoid the onset of a disease phenotype [28-32].

**Figure 1:**
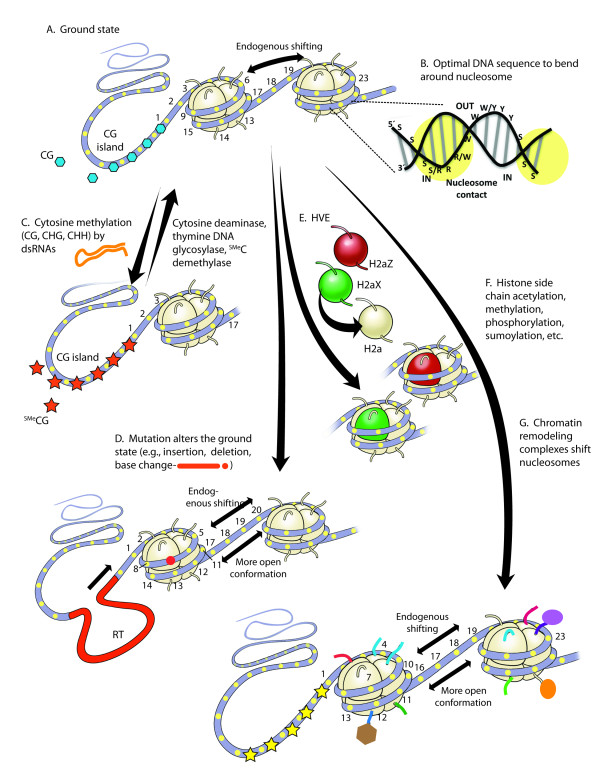
**Summary of relationship between epitype and DNA sequence.A**. Theoretical ground state for a chromatin structure comprised of naked DNA bound to two nucleosomes and an unbound upstream DNA region. Every 10 bp the approximately 2 bp of inward facing surface of the DNA helix has the potential to contact and bind nucleosomal histones (for example, yellow ovals numbered 1 to 23 for region surrounding one nucleosome, see **B**). Each nucleosome has the potential to bind 14 such 2 bp regions. **B**. One 10 bp region of the DNA helix with the consensus ((Y)RRRRRYYYYY(R) provides a bend for optimal nucleosome binding. Nucleotides that provide strong or weak nucleosome binding are indicated (S = strong binding to G or C nucleotides, W = weak binding to A or T nucleotides, R = purine, Y = pyrimidine, IN identifies the surface facing the nucleosome, and OUT the surface facing away from the nucleosome). The strength of nucleosome binding and positioning to 147 bp stretches of DNA appears to be determined by the sum of affinities for 14 small sequences (yellow ovals, same as in **A**). **C**. Double stranded (ds) RNAs (for example, siRNA, piRNA, miRNA) program cytosine methylation for transgenerational inheritance and somatic inheritance in different tissues, while various enzymes remove 5MeC. **D**. Mutations such as single nucleotide polymorphisms (SNPs, red dot) and inserted retrotransposons (RT, red line) may alter nucleosome binding and the stochastic movement of nucleosomes. **E**. Histone variant exchange (HVE) by a subset of chromatin remodeling complexes (for example, SWR1) replaces common core histones (for example, H2A) with specialized protein sequence variants (for example, H2AZ, H2AX). **F**. A variety of histone post-translational modifications (PTMs) of primarily lysine and arginine residues at the N- and C-termini of core histones produce a diverse “histone code” for different nucleosomes. **G**. A large number of chromatin remodeling machines (for example, SWI/SNF, INO80) control nucleosome positioning, often moving nucleosomes in approximately 10 bp increments. Not shown is that the individual epitypes interact with each other to produce complex epitypes. For example, a subset of individuals may contain in their genome a retrotransposons that is targeted by small RNAs, which cause the hypermethylation or hypomethylation of adjacent sequences and alters gene expression (that is, the interaction of **C** and **D**).

This study explores the cause-and-effect relationships among genotype, epitype and phenotype, where the epitype of a single gene or an entire genome is defined as its various cis-linked chromatin structures (Figure 1) [19]. Thus, epitype includes - but is not limited to - chromatin domain structures, such as large DNA loops, the position of all nucleosomes and of subclasses of nucleosomes with particular histone variant compositions (for example, H2A or H2AZ or H2AX), DNA cytosine methylation, and a myriad of histone post-translational modifications (PTMs) [33-35]. By focusing on epitype, we eliminate from consideration several other classes of epigenetic controls such as cell-to-cell communication by morphogens or the inheritance of cell surface patterning [36-40]. Addressing these other epigenetic controls would distract this discussion from a focus on the transgenerational inheritance of chromatin structures.

**Figure 2:**
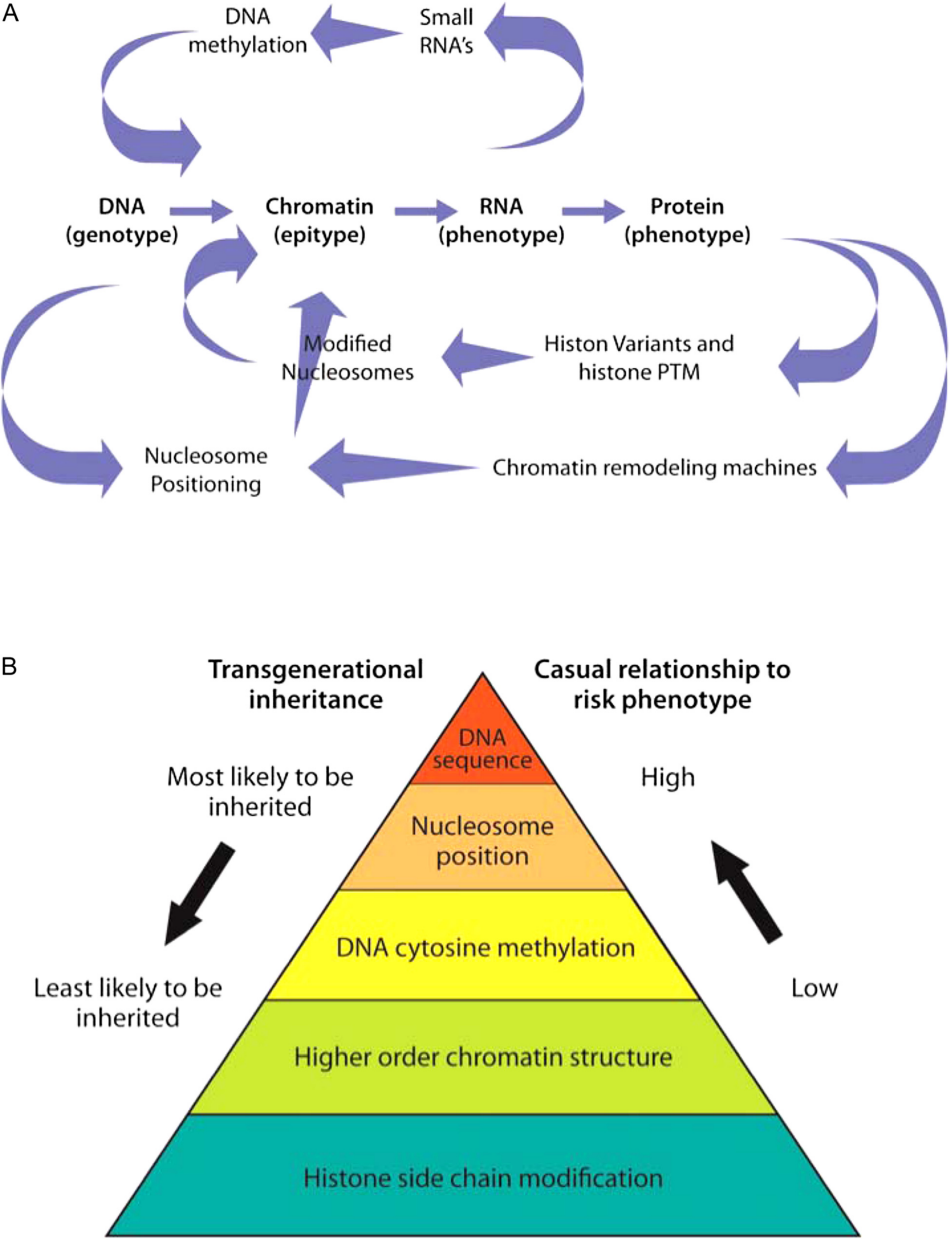
**The relationships among genotype, epitype, and phenotype. A**. The informational relationship and interaction of genotype, epitype and phenotype described in the context of the central dogma of molecular genetics. **B**. A pyramid illustrating the likelihood of different classes of epitypes being transgenerationally inherited and ranking the relative causal relationships of these epitypes to the risk of an aberrant phenotype.

A working hypothesis that emerged from a preliminary examination of the inheritance and evolution of various epitypes [19] is that “genotype predisposes epitype” for most transgenerationally inherited chromatin structures. Only epitypes that are transgenerationally inherited at significant frequencies may contribute to the primary cause of inherited epigenetic risk. Within this hypothesis, epitype and the machinery that alters epitype are modifiers of the central dogma of molecular biology (DNA → RNA → Protein) influencing the activity of DNA and RNA, as shown in Figure 2A. In addition, we will discuss how particular DNA and RNA sequences strongly influence the penetrance of some epitypes and resulting phenotypes. By this view transgenerationally inherited epitypes are not acting at a higher level than or independent of DNA sequence in determining phenotype (for example, RNA and protein expression, disease phenotype).

It will be useful at this point to make the distinction between the transgenerational epigenetic inheritance among parents and offspring and the somatic inheritance between mother and daughter cells within developing tissues and organs [20,23,26,41-45]. The inheritance of epitypes between dividing somatic cells, such as the transmission of a histone PTM [46], is undoubtedly essential to tissue and organ development [47-49] and may be subject to various environmental influences that reveal a phenotype [50]; however, inheritance among somatic cells need not contribute causally to epigenetic inheritance across organismal generations. Again, we are interested herein in identifying epitypes that may be the primary cause of transgenerationally inherited epigenetic risk of acquiring a disease phenotype.

To test this hypothesis our discussion is focused on finding evidence for gene-specific epitypes that supports or rejects cause-and-effect relationships between genotype, epitype and phenotype. Some of the strongest evidence we found, for or against our hypothesis, is summarized in Table 1, and comes from two different research strategies. The first approach (A) examines the penetrance of transgenerationally inherited epitypes that are known to activate or silence the expression of disease related gene(s), which in turn correlate with onset of the aberrant “disease” phenotypes. This direct approach requires the measurement of the frequency of the transgenerational inheritance of causative epitype(s), the relevant gene expression pattern(s), and the aberrant phenotype(s) among related individuals in a population known to be at risk. This method is powerful, produces convincing data, and in several cases reveals the clear contribution of genotype to epitype. But transgenerational measurements are very expensive and time consuming, particularly in the early stages of establishing cause-and-effect relationships to human or agricultural diseases.

**Table 1:** Examples of genes and specific sequences that support or reject the hypothesis that genotype predisposes transgenerationally inherited epitype and phenotype

***Genes/Sequences*affected**	**Genotype contribution**	**Known aberrant epitype/gene expression**	**Phenotype of epimutation or epigenetic change**	**Species**	**Supports/rejects hypothesis**
***A. Direct analysis of trans-generational inheritance***					
*1. RYR1 ryanodine-receptor*	Unknown	Cytosine hypermethylation/silenced	hyperthermia, core myopathies	human	Rejects
*2. MLH1 (Homolog of mismatch repair protein MutL)*	Allele specific silencing	Cytosine hypermethylation/silenced	Colorectal or endometrial cancers	human	Weakly supports
*3. AGOUTI (paracrine signaling peptide)*	Alleles with retrotransposon	Cytosine hypomethylation/activation	Yellow, obese	mouse	Supports
*4. AXIN1-FUSED*	Alleles with retrotransposon	Cytosine hypomethylation, histone acetylation/activation	Axin-fused kinked tail	mouse	Supports
*5. CNR* Colorless Non-Ripening	Native CpG rich region	Cytosine hypermethylation/silenced	Carotenoid synthesis	tomato	Rejects
*6. CYC – cycloidea (transcription factor)*	Native CpG rich region and possible genotype difference	Cytosine hypermethylation/silenced	Floral morphology	*Linaria vulgaris*	Likely supports
7. H3K4Me2 demethylase	None identified	Histone H3 lysine4 dimethylation retained causing gene activation	Germ line immortality	*Caenorhabditis elegans*	Likely rejects
*8. Quantitative epigenetic trait loci (*for example*, many loci)*	DNA DEMETHYLATION1 ddm1/ddm1 restored to DDM1/DDM1	Cytosine re-methylation and re-silencing	Flowering time and plant height	*Arabidopsis thaliana*	Supports
*9. Reprogramming of*^*5Me*^*C by dsRNA*	siRNA, miRNA, piRNA, and other dsRNAs	Cytosine re-methylation and re-silencing	Complex, molecular, and developmental	Arabidopsis, mice	Supports
*10. Somatic cell nuclear transfer*	Genome-wide	Cytosine re-methylation and histone modifications	Embryonic and fetal development	Mice, sheep, pigs, cows	Mostly supports
***B. Indirect analysis using sequence conservation and gene duplication***					
*1.* RRRRRYYYYY repeat throughout the genome	10.5 bp repeats position most nucleosomes	N.M.	N.M.	Diverse animal species	Supports
*2a. Histone H2AZ in >1,000 nucleosomes*	10 bp repeat of G + C and A + T rich dinucleotides	Histone H2AZ variant positioning	Potentiated for expression. N.M.	Yeast, human, Arabidopsis	Supports
2b. H2AZ in *FLC, MAF4, MAF5*	Subfamily of three recently duplicated MADS box genes	Bimodal distribution of H2AZ enriched nucleosomes/activated	Altered flowering time and gene expression	Arabidopsis	Supports
*3. Histone CenH3 in ~100,000 nucleosomes*	10 bp repeat of AA or TT dinucleotides	Histone CenH3 variant positioning	Essential for chromosomal segregation. N.M.	maize	Supports
4. *Blood plasminogen genes (PMGs)*	Cytosine methylation in 208 bp region upstream of four PMG genes	N.M.	Demethylation activates four linked PMG alleles genes in liver. Methylation silences in other organs.	human	Supports
5. *1600 segmental duplications*	Duplicated gene sequences	Several different histone side chain modifications	Duplicate alleles generally silenced relative to active parental allele. N.M.	human	Rejects
6. *HoxD cluster*	Five gene duplicated HOXD genes	Modestly conserved nucleosomal and H3K4Me2 patterns	N.M.	human	Supports
7. *DNA loops and microsatellites*	Concatenated DNA loops and trans-chromosomal contacts	Binding by HMG box proteins to control gene expression	N.M.	mammals	Modestly supports

N.D., no data; N.M., not based on a mutational study.

The second and less direct approach (B) searches out epitypes that are duplicated, as DNA sequences are duplicated, and examines multiple copies of DNA sequence and epitype that have been evolutionarily co-conserved. This approach establishes an unambiguous, and in many cases a statistically significant, correlation of a particular epitype with highly reiterated DNA sequence motifs, and/or examines the conservation of an epitype among duplicated gene sequences. With this strategy, the evolutionary conservation of epitypes among conserved sequences is used as a filter to identify epitypes that were transgenerationally inherited [19,51]. In other words, those epitypes that are widely conserved in their sequence position across the genome or may be shown to evolve by gene duplication within a gene family have almost certainly been inherited through past generations. Again, only epitypes that are transgenerationally inherited have the potential to contribute causally to inherited risk. This second approach simplifies analyses, because the initial screening for likely transgenerationally inherited epitypes may be made within a single genome and in one generation. Conversely, an epitype that is not inherited after gene duplication is less likely to be closely and causally related to phenotype, even if its presence in an allele correlates well with the disease phenotype. Hence, epitypes not inherited via DNA sequence duplications are likely to be poor predictors of inhereted epigenetic risk. The disadvantage of this second genome-centered approach is precisely that it is not focused on finding associated risk phenotypes and during the early stages of analysis we are frequently left with very large datasets describing relationships among epitypes and genotypes without yet knowing correlated pathologies.

#### Direct measurement of transgenerational epigenetic inheritance

Only a handful of studies have succeeded in fully demonstrating that the transgenerational transmission of an epitype produces changes in known target gene expression, which results in a disease or its risk of penetrance (that is, a causal relationship between genotype, epitype, and risk). Two of the best examples from humans concern chromatin structure at the *RYR1* and *MLH1* genes, resulting in muscle myopathies and cancer, respectively. However, the complexity of the data on these two systems highlights the problems that arise when trying to establish such cause-and-effect epigenetic relationships, particularly in humans.

(1) *RYR1:* Genetic mutations causing a loss of expression of *RYR1* function are associated with susceptibility to malignant hyperthermia and congenital myopathies (for example, central core disease, multiminicore disease) [52-54]. However, many individuals with core myopathy disease are known to be heterozygous for a mutant defective *ryr1* allele [54,55]. The epigenetic silencing of the otherwise functional *RYR1* allele appears to account for the loss of functional RYR1 protein expression. For example, among a sampling of 11 patients with the disease, six patients showed tissue-specific silencing of the maternally inherited functional *RYR1* allele, which apparently resulted from cytosine hypermethylation of that allele [56]. Treating skeletal-muscle myoblasts cultured from these patients with 5-aza-deoxycytidine, an inhibitor of cytosine methylation in newly replicated DNA, reactivates the transcription of the epigenetically silenced, but otherwise functional allele. These data strongly support the view that hypermethylation is the primary cause of *RYR1* silencing and onset of an epigenetically determined form of the disease (Figure 2). However, the particular region(s) of DNA in which cytosine residues are methylated to cause gene silencing has not been identified in spite of intense efforts to identify it among three CG islands within the gene. This leaves open the possibility that an epigenetically controlled transacting factor is the causative agent [56]. Thus, for *RYR1* there is not yet a clear causal link between an aberrant genotype, epitype, and the silenced *RYR1* gene expression producing the disease (Table 1).

(2) *MLH1:* The human *MLH1* gene encodes a homologue of the bacterial mismatch DNA repair protein MutL and, hence, *MLH1* is classified as a tumor suppressor. Hypermethylation of DNA cytosine residues and silencing of a particular functional *MLH1* alleles (for example, -93 single nucleotide polymorphism (SNP)) [57], when paired with a dysfunctional mutant allele of the same gene, correlates with relatively young individuals developing tumors of the colorectum or endometrium [27,58]. The tumors and tumor-derived cell lines from individuals with these hypermethylation epimutations fail to express MLH1 protein from this otherwise functional allele [59]. The hypermethylation of the potentially functional *MLH1* allele and its transcriptional silencing is found in most organs and tissues of individuals who also have hypermethylation of this *MLH1* allele in their tumors. Hence, one might expect that this *heritable epimutation* resulted from the transgenerational inheritance of this epitype. However, studies of the children of these individuals generally show loss of hypermethylation and loss of silencing of this *MLH1* allele in the first generation of transmission. Out of several individuals examined, only in one case was the epitype of hypermethylation and silencing inherited through the male parent to the individual with the disease. The *MHL1* silencing phenotype in females with colorectal cancer was associated with a particular CG island centered at −93 bp from the start of transcription in a particular *MHL1* allele containing a SNP, -93 SNP, in this region as illustrated for the more general case in Figure 1C,D [57]. While 5-aza-2'-deoxycytidine will reactivate the silenced allele in cultured cancer cell lines, demethylation is also correlated with a shift in nucleosome position and increased nucleosome density in the promoter region Figure 1A,G [60]. In a very recent study, laser capture microdissection of the ovarian epithelium from ovarian tumors of cancer patients was used to analyze the cell type specific epitype and shows that the hypermethylation of MHL1 is an early somatic event in the malignant transformation of these cells [61]. Cogent to a theme of this article is the fact that the *MHL1* epitypes of aberrant nucleosome position and cytosine methylation appear to be dependent upon the genotype of the epigenetically silenced *MHL1* allele (Table 1). Epimutations of other tumor suppressor genes including *MSH2, MSH6, PMS2*, and *BRCA1* have also been associated with colorectal cancers, but the cause-and-effect relationships with disease are less clear then they are for *MHL1 *[62].

There are considerably more robust examples of the transgenerational epigenetic inheritance from model genetic organisms and wild plants, where it is easier to analyze aberrant epitypes and associated phenotypes through multiple generations**.** A few of the best cases with solid supporting evidence for a relationship between epitype and phenotype will be summarized.

(3) *AGOUTI:* In mice, the secreted AGOUTI peptide functions normally as a paracrine regulator of pigmentation. However, the dominant constitutive expression of the *AGOUTI* gene also targets changes in the hypothalamus and adipose tissues and this aberrant expression causes obesity. Hypomethylated, transcriptionally active dominant epialleles of the *agouti* gene may be maternally inherited through meiosis. Variation in the penetrance of different active epialleles generates a distribution of offspring from abnormal yellow (agouti) obese mice to darker mice with normal amounts of fat [63-65]. Several of the best characterized hypomethylated active and dominant alleles of *agouti* (*Agoutiiapy, Agoutiy, Agoutivy*) that are associated with a high penetrance of the yellow coat color and obesity phenotypes have promoter-containing retrotransposons positioned just upstream of the natural *Agouti* promoter [66,67]. For the best studied alleles, these altered promoter structures are correlated with the hypomethylation of *agouti* and constitutive AGOUTI protein expression. However, a recent detailed examination of the DNA methylation profiles of active and silent alleles suggest that hypomethylation alone may not fully account for the complex ectopic expression of Agouti [18]. Nonetheless, the Agouti examples give reasonable support for the hypothesis (Table 1, Figure 1C,D) that *genotype predisposes epitype* and aberrant phenotype. It would not be surprising to find a shift in promoter nucleosome position resulting from the various retrotransposon insertions contributing to the causative epitype.

(4) *AXIN1-FUSED*: Axin1 is an inhibitor of Wnt (a hybrid of the names for *Wingless* and *Integration1*) signaling that regulates embryonic axis formation in deuterostome animals. In mice, Axin1 is the product of the mouse *Fused* locus. Some murine alleles of *Axin1-fused (Axin1Fu)* show variable and stochastic expression levels, where high expression of a hypomethylated allele correlates with an abnormal kinked tail. Highly penetrant *Axin1Fu* alleles contain an upstream retrotransposon or retrotransposon-mediated DNA rearrangement that alters chromatin structure and contributes to dominant transcript expression [68,69]. An active, highly penetrant mutant allele may be inherited maternally or paternally for multiple generations. Both cytosine hypomethylation and histone acetylation patterns are reported to correlate with increased *Axin1Fu* expression and risk of abnormal tail development [70-72]. The causal relationships between genotype, the DNA methylation epitype, gene expression, and the kinked tail phenotype are supported by the fact that methyl donor dietary supplementation of the mothers, a treatment known to increase DNA methylation, reduced *Axin1Fu* expression and halved the incidence of kinked tails. Conversely, treatment of mice with the histone hyperacetylation agent Trichostatin A increased *Axin1Fu* expression and the frequency of a kinked tail phenotype [72]. This same recent study examining the chromatin from blastocyst stage heterozygous *Axin1Fu/+* embryos shows that dimethylation of lysine-4 on histone H3 (H3K4Me2) as well as acetylation of lysine-9 on histone H3 (H3K9Ac) correlate with penetrant alleles [72]. By contrast, there was no correlation of blastocyst stage cytosine methylation with penetrant alleles. However, both the drug treatments and studies of development after the blastocyst stage only prove the importance of somatic epigenetic inheritance during tail development. Again, it is reasonable to propose that the presence of retrotransposon-mediated changes in DNA sequence, which are present in all the aberrantly expressed Axin1Fu alleles, is the primary cause of the transgenerational inheritance of epigenetic risk. A shift in nucleosome position in penetrant alleles could affect downstream cytosine methylation and histone PTM, resulting in higher *Axin1Fu* gene expression and the kinked tail phenotype. By this view, genotype determines the nucleosomal epitype, which produces other aberrant hypomethylation and histone PTM epitypes, leading to increased gene expression and the novel kinked tail phenotype (Figure 1, Figure 2A, Table 1).

(5) *CNR:* The tomato colorless non-ripening gene *CNR* encodes a homolog of the animal *SQUAMOSA* promoter binding protein (SPB box protein). CNR is essential to normal carotenoid biosynthesis and fruit ripening in the tomato and provides one of the best examples of a stable transgenerationally inherited epitype producing an abnormal phenotype. The natural epialleles of *CNR* in the tomato *Lycopersicon esculentum* contain 18 methylated cytosine residues (5MeCG or 5MeCHG, where H is C, A, or T) in a 286 bp contiguous region [73]. Hypermethylation of this region and silencing of the *CNR* gene leads to colorless tomatoes low in carotenoids (Figure 1C). Because the phenotype is relatively stable, these epialleles were originally mistaken as mutant alleles. The silenced *cnr* epiallele and active wild type *CNR* gene do not have any encoded DNA sequence differences for thousands of base pairs within or flanking this hypermethylated region. Thus, while there is no mutational basis for the change in epitype, the *CNR* gene is potentiated for a stochastic DNA methylation event, because it contains such a large number of strategically positioned cytosine residues in its sequence. While this example supports a link between the *CNR* gene sequence, epitype, and risk phenotype (Table 1), there does not appear to be a particular genotype that predisposes the cytosine hypermethylation epitype. The significant question becomes, once the aberrant epitype is established, how is this hypermethylation epitype stably inherited through the germ line?

(6) *CYCLOIDEA*: The perennial plant in which *CYCLOIDEA* was first identified, *Linaria vulgaris* (Toadflax, Butter and Eggs), normally produces yellow and orange asymmetric flowers composed of three petals of different morphologies. “Mutant” plants are found in wild populations with aberrant abnormally symmetrical “peloric” flowers that are comprised of five evenly arrayed petals of similar morphology. Plants with these aberrant flowers were first characterized by Carl Linnaeus 260 years ago and collected as herbarium specimens [74]. The peloric floral phenotype is produced by the hypermethylation and transcriptional silencing of the gene encoding a transcription factor CYCLOIDEA (CYC) [75]. Inheritance of the recessive peloric floral phenotype and silenced *cyc* epialalele is relatively stable, follows Mendelian segregation and, hence, appeared upon initial investigation to be a normal mutant allele. However, gene silencing always maps to a DNA polymorphic *cyc308G* allele with a single nucleotide polymorphism in an unmethylated region 308 nt downstream of the stop codon and never to the more common wild type *CYC308A* allele. Peloric individuals are homozygous recessive for the *cyc308G* allele with both copies being hypermethylated and completely silenced for RNA expression. Thus, it is reasonable to conclude that *genotype predisposes epitype*, gene silencing, and the peloric phenotype (Table 1, Figure 1C,D).

(7) *Histone H3K4Me2 demethylase erases epigenetic memory in each generation:* A number of histone PTMs such as H3K4Me2 are acquired during transcription and are associated with active genes [76]. These epigenetic marks are removed at different stages in development by an H3K4Me2 demethylase, known as LSD1 in humans and SPR-5 in *Caenorhabditis elegans* (Figure 1F). Removal of the H3K4Me2 epitype prior to meiosis by SPR-5 in *Caenorhabditis elegans* is essential for maintaining an immortal germline [77,78]. Within two-dozen generations of worms acquiring the recessive null genotype these *spr-5* mutants have a brood size several-fold lower than wild type, with 70% of the worms being fully sterile. Homologs of LSD1 (SPR-5) are found throughout the four eukaryotic kingdoms and a number of these genes are known to be essential for normal organismal development [79-81]. The unmodified H3K4 epitype is essential and retention of the histone PTM causes aberrant development. However, there is as yet little evidence that this particular histone PTM epitype is normally preserved through meiosis or that genotype plays any role in determining the H3K4Me2 epitype at any particular locus (Table 1).

(8) *Inheritance of quantitative epigenetic trait loci.* Two separate genome-wide epigenetic studies demonstrate that multi-generational inheritance of complex traits such as flowering-time, plant height, biomass, and bacterial pathogen resistance behave as quantitative epigenetic trait loci in *Arabidopsis thaliana *[22,82,83]. These studies used two independently derived sets of recombinant inbred lines (RILs), where one of the founding parents was a recessive null for one of two known genes necessary for DNA cytosine methylation. For example, one study begins with a fourth generation plant homozygous defective *ddm1/ddm1* that is highly compromised in a number of phenotypic traits due to DNA hypomethylation. *DECREASED DNA METHYLATION1* (DDM1) is a Swi2/Snf2-like DNA-dependent ATPase chromatin remodeler required for most DNA cytosine methylation. The *ddm1/ddm1* line was backcrossed to wild type, and this heterozygous F1 *ddm1/DDM1* was backcrossed to wild type again and screened to obtain hundreds of separate DDM1/DDM1 lines. These lines were selfed to establish hundreds of epiallelic recombinant inbred plant lines (epiRILs) [22]. For several generations, approximately 30% of the *DDM1/DDM1* epiRILs displayed aberrant morphological phenotypes affecting flowering time and plant height, among other phenotypes. They assayed 22 epiRILs for the methylation of 11 candidate genes that are normally cytosine hypermethylated, but are hypomethylated in *ddm1*. Six alleles showed partial remethylation and five alleles were completely remethylated producing the identical complex epitype for this later gene set to wild type. Control genes that were previously unmethylated remained unmethylated.

In one particular example, Johannes and colleagues [22] followed the methylation sensitive *FWA* gene, for which the ectopic expression of the hypomethylated epiallele in *ddm1* parental plants produces strong late flowering phenotypes [84]. All of the 22 randomly selected epiRILs were now normally methylated at FWA and flowered at normal times. However, when they examined three extremely late flowering lines from among the population of hundreds of epiRILs (that is, plants that flowered after more than 48 days versus 33 days to flowering in wild type) these epiRILs were almost completely hypomethylated at *FWA* and expressed high levels of *FWA* transcripts, accounting for their phenotype. Hence, out of hundreds, only a few of the epiRILs escaped from the remethylation of *FWA,* when DDM1 was restored.

In summary, aberrant DNA methylation epitypes at many loci and the resulting changes in downstream molecular and developmental phenotypes appear to be transgenerationally inherited. Most genes regain wild type methylation patterns and phenotypes within a few generations and the restoration appears to be sequence specific. Hence, the genetic machinery necessary for the *de novo* remethylation of these completely unmethylated loci is encoded in the Arabidopsis genome and remethylation did not require hemi-methylated DNA templates to be newly inherited. These data suggest that genotype predisposes this global cytosine methylation epitype.

(9) *Reprogramming of DNA cytosine methylation by double stranded dsRNAs.* The 5´-methylation of DNA cytosine residues occurs in three sequence contexts: 5MeCG, 5MeCHG and 5MeCHH (Figure 1C). A number of DNA methyl-transferases (DMTs) are known to methylate DNA cytosine in the 5´ position. DMT1 efficiently propagates hemimethylated symmetrical CG sequences and, hence, the somatic inheritance of islands of 5MeCG hypermethylation that may lead to gene silencing is not hard to explain. However, DNA methylation of all types is predominantly erased (that is, 80 to 90% loss of methylation) in germ line cells in the embryos of both plants and animals [85-87]. Hence, the reprogramming of CG, CHG, and CHH methylation and a mechanism for transgenerational inheritance of these epitypes has been of intense interest in recent years [88,89]. To simplify the discussion of the gene-specific DNA cytosine remethylation and subsequent inheritance of methylation, Richards [90] introduced three working categories: obligate, facilitative, and pure DNA methylation.

Epialleles in heterochromatic DNA that display obligate DNA cytosine methylation always remain methylated due to the presence of large numbers of transposable elements in various orientations producing dsRNA that promote a strong RNA interference response and adjacent target gene remethylation [91]. Genes within or closely adjacent to the centromer are good examples of obligate epialleles. Axin1Fu and AgoutiAy are typical examples of facilitative epialleles, because the presence of an upstream change in DNA sequence facilitates a seemingly stochastic epigenetic variation in methylation and phenotype. Because the wild type loci for these alleles lack an altered promoter element there is seldom any variation in the cytosine methylation epitype at the wild type loci. Pure epialleles are defined as those showing variation in cytosine methylation without a known genotypic cause and appear to be examples of *de novo* DNA cytosine methylation. If pure epialleles are truly independent of genotype, then they stand as strong evidence against our hypothesis. The well studied hypermethylation and silencing of wild-type *CNR* and *RYR1* alleles fit the definition of pure epialelles. Schmitz and colleagues [92] examined the complete methylome of 100 Arabidopsis lines propagated for 30 generations by single seed descent from a single parent. They observed that CG ↔ 5MeGC single methylation polymorphisms (SMPs) occurred at a 10,000-fold increased frequency per generation over the DNA base mutation rate, which they also measured (Figure 1D). While CG SMPs occurred primarily within gene bodies, large numbers of CHG and CHH SMPs occurred in flanking regions. Thus, novel inherited SMPs are generated at high frequencies and, if this remethylation is independent of DNA sequence, then pure epialleles are common.

One relevant question for this discussion is the following: are ostensibly pure epialleles truly independent of genotype, or are they simply facilitative epialleles for which we have not yet identified the associated cis- or trans-acting genes making dsRNAs that program inherited CG, CHG and CHH methylation epitypes? There is recent evidence supporting the latter interpretation that we now summarize.

Despite being generated through slightly different mechanisms, many classes of small RNAs (for example, siRNA, miRNA, piRNA) are known to template the remethylation of cytosine in different sequence-specific contexts (Figure 1C) for the transgenerational inheritance of gene silencing and or activation [89,93]. This general mechanism for reprogramming using different classes of small RNAs appears ancient in that it is found in all four eukaryotic kingdoms. These RNAs facilitate the remethylation of appropriate CG, GHG, and CHH sequences. But these data began to raise the question: does remethylation occur on a global genome-wide scale? To address the scope of remethylation, Teixeira and colleagues [94] examined the remethylation of numerous transposable element loci in DDM1/DDM1 epiRIL plants that had descended from an essentially unmethylated *ddm1/ddm1* plant backcrossed to wild type. Those loci that were remethylated after a few generations in the epiRILs contained cytosine rich gene sequences that were highly complementary to the sequence of siRNAs. Those loci with similar cytosine rich composition for which they could not identify complementary siRNAs remained hypomethylated. siRNAs attract RNA interference (RNAi) and DNA methylation machinery to complementary DNA sequences and thereby template sequence-related DNA methylation [95]. This shows that RNAi mechanisms are essential for the proper remethylation of much of the Arabidopsis genome. These and other data make it clear that, for a large number of repetitive elements in yeast, plants, and animals, the matching genotypes of structural genes and small RNAs predict a cytosine methylation phenotype. However, the study of Teixeira and colleagues [94] raises further questions about the biology, regulation, and timing of cytosine remethylation for both transgenerational and somatically inherited epitypes. Recent evidence suggests that in both plants and animals “nurse cells” may transfer hundreds of undefined small RNAs to adjacent egg or sperm germ cells to reprogram cytosine methylation [88,89,93]. For example, in mice in which 80% to 90% of the germline DNA methylation is erased for single copy genes at approximately day 11.5 of embryo development (E11.5). Remethylation of sperm DNA occurs in the embryo at approximately E16.5 and is significantly directed by populations of 24 to 30 nt long piRNAs produced in adjacent cells in the pro-spermatogonia [96-98]. The identities of most of the plant and animal small RNAs transferred to developing germ cells are not yet known, but there is the real potential that large populations of RNAs may account for most transgenerational remethylation and perhaps even the apparent *de novo* methylation described by Schmitz and colleagues [92]. Appropriately positioned target sequences in these epialleles and thousands of expressed small RNAs would have to be inherited together for genotype to predispose the transgenerational inheritance of the global DNA methylation epitype.

(10) *Reprogramming epitype during somatic cell nuclear transfer.* In most of the above examples, genotype determines the likelihood, but not the certainty, of particular epitypes and phenotypes being displayed, because the same DNA sequence may be flexibly reprogrammed into many different chromatin conformations. It is fundamental to epigenetics that as cell types differentiate the same DNA sequence may display multiple epitypes and some epitypes may be more or less stable than others. An interesting example of a variety of epitypes descending from one genotype comes from research using somatic cell nuclear transfer (SCNT) to produce identical or genetically modified laboratory and farm animals. SCNT is achieved by transplanting a somatic cell nucleus into a functional embryonic cell capable of forming a viable organism. This technology has met with modest success, generating cloned mice, rabbits, pigs, sheep, cows and more, but the efficiency of obtaining viable healthy offspring is low. Even if genetically modified embryos are established in surrogate mothers, developmental abnormalities and spontaneous abortions are common. A major limitation to obtaining relatively normal full-term development appears to be variations in epigenetic reprogramming of the transplanted nucleus [99-102]. The field of regenerative medicine faces similar problems with epigenetic reprogramming when trying to establish genetically altered lines of induced pluripotent stem cells by SCNT - for example, by transferring a somatic cell nucleus into an oocyte [103,104]. Without prior knowledge of the successes in producing cloned animals by SCNT, one would not necessarily expect that the new nuclear environment should correctly reprogram the donated nucleus. A known source of the reprogramming problem in the animal cloning field is that the transferred nucleus frequently loses a significant fraction of its DNA cytosine methylation and nucleosomal histone side chain methylation and acetylation relative to the more modified epitype of nuclei in native embryonic cells (Figure 1C,F) [105-109]. However, the surprising fact remains that some relatively healthy animals resembling the nuclear donor are obtained via SCNT and that genetic and epigenetic totipotency of the donor nucleus is re-established in the viable offspring. For appropriate reprogramming to take place on a genome-wide scale the donor DNA sequence must have the capacity to interact with the embryonic cellular environment and determine, albeit at low frequency, an epitype(s) compatible with full-term development. These results support the idea that during SCNT the donated DNA sequence predisposes much of its own epigenetic reprogramming (Table 1).

#### Evolutionary co-conservation of DNA sequence and chromatin structure filters out transgenerationally inherited epitypes

If genotype pedisposes epitype then a reasonable corollary is that some transgenerationally inherited chromatin structures should align with particular DNA sequence motifs and be passed on to duplicate gene copies. In this model, the range of possible epitypes for a sequence would evolve by gene duplication and mutation in parallel with genotype [19,51]. Rapidly evolving epitypes might only be conserved and identifiable among very recently duplicated genes examined among a limited number of related cell types or when examined statistically in comparisons of large numbers of aligned sequences, while slowly evolving highly conserved epitypes might be found among anciently duplicated genes and descended from a common ancestral protist sequence.

(1) *Short DNA sequence repeats such as RRRRRYYYYY determine the bending and positioning of DNA around the nucleosome.* More than 30 years ago, Trifonov and his colleagues [110,111] presented the case that gene sequence is fundamentally important to nucleosome positioning. He argued that the necessary high degree of bending of DNA as it wraps twice around and binds the nucleosome would be favored by particular 10.5 bp repeat sequences of approximately 5 purines (R) followed by 5 pyrimidines (Y) (*RRRRRYYYYY*) (Figure 1B), or the inverse of this sequence, *YYYYYRRRRR*. He also found a good correlation for 10 bp repetitions of the dinucleotides GG, TA, TG, and TT in the modest compilation of 30,000 bp of DNA sequence from different eukaryotes available at that time.^a^ Within the 10 bp motif these dinucleotides were proposed to help position nucleosomes. The statistical concept was a bit counterintuitive and slow to gain acceptance, because it was hard to reconcile the functional demands of sequences encoding proteins and regulatory regions with the proposed special sequence demands of nucleosome interaction. Recently, with access to nearly unlimited numbers of nucleosome-delimited 147 bp DNA sequence fragments and more advanced computational methods, it has become very clear that 14 repetitions of the 10.5 bp repeat sequences *Y-RRRRRYYYYY-R* or *R-YYYYYRRRRR-Y* are statistically favored for nucleosome positioning. Regional differences in GC compositions in the genome favor particularly skewed repeats such as *T-AAAAATTTTT-A* or *C-GGGGGCCCCC-G *[112,113]. These consensus sequences are based on a statistical argument, and at the genome level any one dinucleotide such as *AA* or *GG* is seldom found in a particular position in the 147 bp repeat more than 30% of the time [114]. Because the inward facing helix of any one 147 bp of nucleosomal DNA fragment has 14 chances to contact the core of nucleosomal proteins, this mechanism requires only several correctly positioned dinucleotides contacting the nucleosome to give sequence specificity to nucleosome positioning. Hence, there is in fact little conflict with conserved coding and regulatory sequences and the sequence constraints of nucleosome positioning. Furthermore, the most common classes of ATP-dependent chromatin remodeling machines, switch/sucrose nonfermentable (SWI/SNF) and imitation switch (ISW)2, move DNA in approximately 9 to 11 bp increments over the surface of a nucleosome, consistent with the importance of 10.5 bp repeats in nucleosome binding [115]. These data strongly support a model where genotype predisposes possible nucleosome position epitypes. More particular support for this argument comes from examining the sequences for subsets of the nucleosomal DNA population binding nucleosomes containing histone variants H2AZ and CENH3.

(2a) *The geneome-wide positioning of H2AZ nucleosomes*. The histone variant H2AZ and likely other histone variants are inserted into assembled nucleosomes by histone variant exchange complexes (HVE) such as SWR1 (Figure 1E). Albert and colleagues [116] precisely aligned the sequences of thousands of 147 bp yeast nucleosomal DNA fragments enriched for histone variant H2AZ. Their data show conclusively that H2AZ nucleosome positioning on a genome-wide scale is strongly influenced by dinucleotide repeat patterns spaced 10 bp apart in the DNA sequence (Figure 1A,B). In particular, GC-rich dinucleotides are on the inside as the DNA helix wraps around the nucleosomal protein core, and AT-rich dinucleotides are on the outside. The preference for these nucleotide pairs at each of their 14 possible positions within any 147 bp nucleosomal fragment is only about 2 to 9%, and therefore any single nucleosomal fragment sequence is likely to vary significantly from the statistical consensus. However, it is clear that the H2AZ nucleosome position is determined by the overall pattern in the DNA sequence and, hence, H2AZ nucleosome position will be conserved following gene duplication. Similar results were obtained for genome-wide positioning of all nucleosomes from humans and Arabidopsis [117] and subsets of human nucleosomes specific to certain classes of genes [118]. In the total 147 bp nucleosomal fraction from Arabidopsis and humans, an AT-rich dinucleotide repeat is spaced every 10 bp and out of phase by 5 bp with a GC-rich dinucleotide repeat.

(2b) Further support for the concept that DNA sequence positions H2AZ nucleosomes comes from a comparison of duplicated genes in Arabidopsis. A single peak of H2AZ enriched nucleosome(s) is found at the 5´ end of nearly half of all plant, animal, and fungal genes that have been examined [116,119-121]. In Arabidopsis, three related MADS box genes that regulate flowering time require normal H2AZ for full expression. In wild-type cells, all three MADS box genes show a striking bimodal distribution of H2AZ deposition, with peaks of H2AZ histone-containing nucleosomes at their 5´ and 3´ ends [122]. This pattern is quite distinct from the single 5´ spike of H2AZ observed for other MADS genes in humans, *Arabidopsis*, and yeast. These three genes are estimated to have diverged from a common gene ancestry in the eudicot plant lineage in the last 100 million years and stand alone in their own distinct clade, among more than 100 other MADS box genes in Arabidopsis that do not have a bimodal distribution of H2AZ nucleosomes. These data are consistent with the bimodal distribution of H2AZ being inherited following gene sequence duplication from an ancestral MADS gene [19].

(3) *The genome-wide positioning of CENH3 centromeric nucleosomes.* Recent experimental evidence demonstrates that CENH3 enriched centromeric nucleosome positions are determined by DNA sequence. Animal and plant centromeres are composed of a diverse variety of retroelements and repetitive satellites that generally appear unrelated in their DNA sequences. Numerous earlier studies of centromere and neocentromere sequences concluded that a distinct conserved DNA sequence was not essential to centromere activity. However, a very recent analysis of 100,000 centromeric histone CENH3 enriched nucleosomal DNA fragments from maize suggests that a 10 bp repeat of AA or TT dinucleotides contributes to determining the positioning of centromeric nucleosomes [123]. The CENH3 nucleosome specific sequence was not revealed until the 147 bp micrococcal nuclease protected DNA sequences were precisely aligned. The preference for AA or TT nucleotide pairs at each of the 14 positions within a typical 147 bp nucleosomal fragment was statistically significant. The likelihood of finding one of these dinucleotide pairs at any of the potential contact points ranges from 13% to 60% above the frequency at which other dinucleotides are found. Thus, CENH3 enriched nucleosomes are positioned by a variation on what is shown in Figure 1B, where the inward facing DNA base pairs that bind are generally AA or TT and would be classified as weak binding. This would indicate that any single centromeric nucleosomal sequence may vary significantly from the statistical consensus for these nucleotide pairs. In this way, a subset of retroelements that are seemingly unrelated in sequence using standard sequence alignment methods may contain suitable sequence repeats that position centromeric nucleosomes.The human and Arabidopsis genomes each encode more than a dozen histone protein sequence variants for each of three classes of histones, H2A, H2B, and H3. Within each class a few subclass variants are easily identified as predating the divergence of plants, animals, and fungi from their more recent protist ancestors. Thus, it is reasonable to speculate that distinct DNA sequence patterns evolved in concert with each histone variant subclass to provide complex patterns of nucleosome positioning. If true, then DNA sequence would be responsible for the transgenerational positioning of most classes of nucleosomes.

(4) *Cytosine methylation in the human plasminogen gene family.* In an attempt to show that epitypes and associated phenotypes can evolve by gene duplication and divergence, Cortese and colleagues [51] compared promoter CG methylation patterns among the four duplicated gene members of the approximately 35-million-year-old human plasminogen (*PLG*) precursor gene family, encoding blood-clotting factors found only in hominids. Cytosine DNA methylation patterns are well conserved among seven CG sites located −171 to −378 nucleotides upstream from the start of transcription within all four *PLG* gene promoters (similar to Figure 1A,C). In liver, where transcripts for all four genes are expressed, one allelic copy of each gene pair is almost completely unmethylated at all seven sites. In heart muscle and in skeletal muscle, where the four *PLG* genes are turned off, nearly 100% of the seven sites are fully cytosine methylated on both alleles for all four genes. In other words, promoter cytosine methylation silences all gene copies in the two nonexpressing tissues examined, while hypomethylation of one copy of each *PLG* gene activates their expression in liver. The *PLG* data support the generational inheritance and conservation of the cytosine methylation epitype following gene duplication for recently duplicated genes that are co-expressed. Cortese and colleagues [51] also compared promoter CG methylation patterns among several members of the much older human T Box (TBX) gene family in which the most gene duplications date back 300 to 600 million years. No evidence was obtained for conserved CG methylation patterns among any pair-wise comparison of *TBX* genes. Perhaps because the *TBX* genes are differentially expressed and the divergence events between genes are much more ancient, the lack of conserved CG methylation patterns is to be expected.

(5) *Histone side chain modifications in human segmental sequence duplications.* Barski and colleagues [124] published a ground-breaking genome-wide study on sequence specific location of 23 histone PTMs and a few other epitypes in purified human CD4+ T cells. From this dataset, Zheng [125] examined 14 distinct patterns of histone PTM in nucleosomes from 1,646 relatively recent (that is, less than approximately 25 million-year-old) segmental chromosome duplications (SDs). They found no significant evidence for the inheritance of these histone modifications between the original and derived loci. Specifically, the duplicated copy did not inherit the parental pattern of histone side chain methylation or acetylation (Figure 1F). Moreover, inheritance appears to be distinctly asymmetric for some of the modifications, such that there is a strong statistical bias toward histone methylation of one gene copy for each SD and not the other copy, beyond what might have occurred at random. Many of the asymmetrical histone modifications correlate with gene activation and repression, suggesting that active genes in the parent sequence are silenced in the duplicated loci, and *visa versa*. These data imply that histone PTM epitypes may not be the direct transgenerationally inherited “cause” of the phenotypes with which they are associated. Thus, these data on histone PTM epitypes at SDs do not support our working hypothesis. If these results are supported by more experimental studies, it will not mean that histone modifications are not useful epitypes for predicting risk, but that they may be further from the inherited cause of epigenome-induced pathologies than other epitypes such as nucleosome position and cytosine methylation. Histone PTMs are indeed important to somatic inheritance and development [46,126].

(6) *Nucleosome positioning and H3K4Me2 modifications in the HOXD cluster.* There are six genes at the *HOXD* gene cluster (that is, HOXD13, 11, 9, 8, 4, 3) covering approximately 100,000 bp on human Chromosome 2. In human sperm, there are one or two spikes of general nucleosome occupancy and H3K4Me2-enriched nucleosome occupancy within each of the promoters of these genes, whereas the approximate 100,000 bp of 5´ flanking region is relatively free of nucleosomes [127] (Figure 1A,F). Because nucleosome positioning was performed using microarrays, the sequence specificity of H3K4Me2-enriched nucleosomes among these *HOXD* promoters cannot be determined from these data or compared to the results from Barski and colleagues [124] who did not find sequence specificity for histone H3K4Me2-enriched nucleosome binding. These results showing the conserved positioning of nucleosomes in *HOXD* promoters in human sperm are similar to those for H2AZ-enriched nucleosomes among the *FLC*-related *MADS* genes in Arabidopsis shoot tissue [122].

(7) *Higher-order chromatin structures.* Genes and regulatory sequences that are narrowly or widely spaced on a chromosome may interact productively through higher order chromatin structures such as solenoids, small and giant loops, and minibands [128-130]. For example, small concatenated DNA loops may be formed by re-association of the single strands of the poly (CA)-poly (TG) microsatellite at their base [131]. These small loops appear to impact the control of gene expression via binding to HMG-box proteins [131,132]. There is mounting evidence that interactions of distant intra- and inter-chromosomal domains provide epigenetic mechanisms to maintain specialized gene expression states [133-135]. Hence, the potential exists that higher order structures contribute to epigenetic control and are determined in part by DNA sequence.

### Summary from direct and indirect analyses of epigenetic inheritance

An examination of several examples of the direct transgenerational inheritance of epitype and the epitypes of duplicated and/or conserved DNA sequences revealed the complexities of determining cause-and-effect relationships among genotype, epitype and phenotype. However, in balance, there are robust experimental data supporting the hypothesis that “genotype predisposes epitype,” for some epitypes (Table 1). In particular, it is becoming clear that a large fraction of, if not all, cytosine methylation is determined by gene sequence and the presence of paired sequence-specific complementary small RNAs that direct their transgenerational remethylation. Similarly, based on the sequences of H2AZ and CENH3 enriched nucleosomal fragments, nucleosome position appears strongly influenced by DNA sequence (Figure 1A,B,C). However, there is little evidence suggesting that DNA sequence determines the position of any of more than 20 different classes of histone PTM enriched nucleosomes (Figure 1F, Table 1).

Based on this analysis, it is worth ranking the utility of various classes of epitype in estimating epigenetic risk. A risk pyramid linking the relationships of genotype and epitype with epigenetic risk phenotype is shown in Figure 2B. DNA sequence is placed at the apex, as the primary cause of inhereted epigenetic risk. This is followed by nucleosome position that appears to be directly dependent upon 10 bp repeats in DNA sequence and DNA cytosine methylation that is highly dependent upon *cis*-acting CG, CHG, and CHH sequences in the target gene and the sequence of trans-acting small RNAs. However, while histone PTM may be strongly correlated with epigenetically controlled phenotype, there is no evidence that any histone PTM is causal to transgenerationally inherited risk. Histone PTM epitypes may represent the effect of other epigenetic and genetic controls and may be principally important to somatic inheritance of epigenetic controls. The clear relationship between novel genotypes and many of the most robustly characterized inherited epitypes of nucleosome position and cytosine methylation is a recurrent theme in the literature of the most thoroughly studied genes under epigenetic control. This suggests that human and animal therapeutic treatments or plant and animal genetic breeding strategies that address harmful meiotically inherited epitypes should consider the possibility that there are genotypic causes predisposing these epitypes. If, for example, the environment of a developing somatic tissue (for example, obesity, stress, nutrients) is influencing RNA sequence directed cytosine remethylation and gene silencing, drugs targeting downstream histone PTM epitypes of that gene may be less effective than ones addressing remethylation. Strategies directed at controlling gene expression by altering histone PTM epitypes may be useful if they target the gene or genes producing the disease’s phenotype. Finally, the undeniable influence of genotype on epigenetic controls leading to deleterious phenotypes has to be taken into account in a consideration of epigenetic risk, even if it confounds many current, working definitions of epigenetics.

### Defining epigenetics

We’ve summarized direct and indirect evidence that genotype predisposes epitype and that epigenetic controls are strongly influenced by DNA and RNA sequences (Figures 1 and 2). Our hypothesis and these supporting data may be viewed as contrary to some of the widely stated precepts of epigenetics. For example, Riggs and colleagues defined epigenetics as “*the study of mitotically and/or meiotically heritable changes in gene function that cannot be explained by changes in DNA sequence” *[34,136]. A rephrasing of this statement as “*the study of mitotically and/or meiotically inherited changes in gene function that cannot be explained by the classical central dogma of molecular genetics”* (Figure 2A) provides a working definition that is quite consistent with our deliberations*.* In David Nanney’s seminal article describing epigenetic control systems, he states “*The term "epigenetic" is chosen to emphasize the reliance of these systems on the genetic systems*” and goes on to say “*epigenetic systems regulate the expression of the genetically determined potentialities*” [39]. Nanney’s definitions of epigenetics are completely consistent with genotype predisposing inherited epitype, and with epitype modifying gene expression and risk phenotype.

### The influence of DNA sequence on epigenome-induced pathologies points a way forward

Understanding that genotype predetermines many inherited epitypes suggests a few useful strategies and concerns as we try to address epigenome-induced pathologies. First, we are in a better technical position than ever before to determine the influence of genotype on epitype. New rapid DNA sequencing and DNA bead array methods for identifying SNPs and 5MeC residues combined with a wide selection of treatments to chromatin (for example, ChIP, bisulfite, micrococcal nuclease) allow us to quantitatively determine the precise genome-wide sequence-specific positioning of every nucleosome, methylated cytosine residue, and dozens of distinct histone PTMs in a genome. These epitypes may be correlated with the risk of cancer, behavioral disorders, pathogen susceptibility, or the role of aging and environmental factors on risk, as examples. The lower costs of genome-wide approaches is enabling the epitypes of larger populations of humans, laboratory animals, and plants to be examined in order to identify the epigenetic causes of complex diseases such as obesity, lupus, or pathogen susceptibility [137-140]. Second, we are in a position to develop batteries of gene-specific epigenetic biomarkers for DNA methylation epitypes that are clearly associated with disease risk and may be predictive of the penetrance of pathology. For example, this is currently being done for systemic lupus erythematosus, myeloid leukemia, and breast cancer [138,141-143]. However, new technologies are needed if we are also to use nucleosome position and histone PTM epitypes as inexpensive epigenetic biomarkers for screening populations. Third, because the development of each plant and animal cell type in an organ system is under strong epigenetic control, it is essential that we examine epitypes in distinct cell types within organs. Most current epigenetic studies examine mixed cell types such as are present in whole organs and tissues (for example, blood, tumor, hypocampus, skeletal muscle, plant shoots or roots), wherein cell type-specific epitypes are blurred due to variation of epitypes among developmentally distinct cell types. For example, several orders of magnitude more statistically significant relationships were obtained between the cytosine methylation epitype of various genes with lupus when CD4+ T cells were examined as compared to the data obtained from mixed populations of white blood cells [138,144]. Technologies have been developed to access cell type-specific epitypes, including laser cell capture micro-dissection, fluorescent activated cell sorting (FACS) of dissociated fluorescently tagged cells, and the isolation of nuclei tagged in specific cell types (INTACT). These technologies enable the more precise determination of epitypes within individual cell types as has been shown for CD4+ T cells, primordial germ cells, ovarian epithelium, retinal cones, and plant root epithelial trichoblasts and atrichoblasts [61,124,145-148]. Fourth, therapeutic approaches to human epimutations that increase the risk of pathology, or plant breeding strategies to address epigenetic susceptibility to stress or disease, need to consider that molecular mechanisms may be obscurely hidden in DNA sequence motifs and/or the sequences of small RNAs that are imperfectly matched with their target genes (Figure 1). Current basic research is laying the course for using small RNAs to direct transcriptional gene silencing by promoter DNA methylation for therapeutics and crop improvement. For example, siRNA transgenes have been used for the methylation-based transcriptional silencing of the *Heparanase* gene in human cancer cells in culture [149] and to elucidate the mechanisms of small RNA-based transcriptional silencing in plants [150,151]. Unless we can develop therapeutic approaches, identifying genotypic influences on epigenetic risk may only add more diseases to the list of thousands for which we know the cause, but have no known cure. However, taking the numerous advances in epigenetics research altogether, it is reasonable to propose that during the next two decades effective therapeutic treatments will follow the dissection of the molecular mechanisms by which genotype and epitype interact to produce disease pathologies.

## Conclusion

There is substantial evidence that altered epigenetic controls contribute to a variety of diseases ranging from cancer and developmental malformations to susceptibility to various forms of biotic and abiotic stress. We reviewed experimental genetic, epigenetic, cell biological, and biochemical data surrounding the transgenerational inheritance of several examples of well studied epigenome-induced pathologies and the contribution of conserved DNA sequence motifs to epitype. The preponderance of evidence suggests that genotypes predispose epitypes for most chromatin structures that are transgenerationally inherited and this relationship contributes to the penetrance of epigenetically controlled diseases. Genotypes influencing inherited epigenetic risk are often obscurely encoded in DNA sequence and small RNAs. Furthermore, the remethylation of DNA cytosine residues may only be reprogrammed at particular times in development and only in particular tissues such that a special effort may be required to identify and characterize these mechanisms. Some of the best characterized examples that were discussed herein suggest we are only just beginning to understand the molecular biology behind inherited epigenome-induced disorders. Finally, the paths to effective therapeutic development or to lowering epigenetic risk will be easier to trace out once we understand the mechanisms by which genotype predisposes epitype for a particular disease.

## Endnote

^a^Trifinov did not have nucleosome specific DNA sequence data available 30 years ago.

## Abbreviations

CNR, Colorless non-ripening; CENH3, Centromeric histone H3; CYC, CYCLOIDEA; DDM1, DECREASED DNA METHYLATION1; DMT, DNA methyl-transferase; dsRNA, Double stranded RNA; epiRIL, Epiallelic recombinant inbred plant line; FACS, Fluorescent activated cell sorting; FWA, FLOWERING WAGENINGEN; H3K4Me2, Histone H3 dimethylated at lysine4; H3K9Ac, Histone H3 acethylated at lysine-9; HVE, Histone variant exchange complexes; INTACT, Isolation of nuclei tagged in specific cell types; ISW, Imitation switch; MLH1, MutL Homolog 1 gene; piRNA, Piwi-interacting RNA; PLG, Plasminogen; PTM, Post-translational modification; R, Purine; RIL, Recombinant inbred line; RNAi, RNA interference; RYR1, Skeletal-muscle ryanodine-receptor gene; SCNT, Somatic cell nuclear transfer; SD, Segmental chromosome duplication; siRNA, Small inhibitory RNA; SMP, Single methylation polymorphism; SNP, Single nucleotide polymorphism; SPB box protein, SQUAMOSA promoter binding protein; SWI/SNF, Switch/sucrose nonfermentable; TBX, T Box; Wnt, Is a hybrid of the names for Wingless and Integration1; Y, Pyrimidine.

## Competing interests

The authors declare that they have no competing interests.

## Authors’ contributions

RBM proposed the hypothesis that drove the content of the manuscript and wrote most of the manuscript. KJM helped with the conception of the manuscript and topics, designed the epigenetic risk pyramid, and contributed to the writing and fact checking. Both authors read and approved the final manuscript.
